# Understanding socio-sexual networks: critical consideration for HIVST intervention planning among men who have sex with men in Kenya

**DOI:** 10.1186/s12889-022-12901-x

**Published:** 2022-03-21

**Authors:** Lisa Lazarus, Ravi Prakash, Bernadette K. Kombo, Matthew Thomann, Kennedy Olango, Martin K. Ongaro, Samuel Kuria, Memory Melon, Helgar Musyoki, Souradet Shaw, Parinita Bhattacharjee, Robert Lorway

**Affiliations:** 1 Institute for Global Public Health, Rady Faculty of Health Sciences, University of Manitoba, R070 Med Rehab Bldg, 771 McDermot Ave, Winnipeg, Manitoba R3E 0T6 Canada; 2 India Health Action Trust, Bangalore, India; 3 Department of Anthropology, University of Maryland, College Park, MD USA; 4 Men Against AIDS Youth Group, Kisumu, Kenya; 5 HIV and AIDS People’s Alliance of Kenya, Mombasa, Kenya; 6 Mamboleo Peer Empowerment Group, Kiambu, Kenya; 7 Partners for Health and Development in Africa, Nairobi, Kenya; 8 Ministry of Health, National AIDS and STI Control Programme, Nairobi, Kenya

## Abstract

**Background:**

HIV self-testing (HIVST) has emerged as a way of reaching individuals who may be less likely to access testing, including men who have sex with men (MSM). Understanding the social networks of MSM is key to tailoring interventions, such as HIVST, for particular locations.

**Methods:**

We undertook a socio-sexual network study to characterize and identify patterns of connection among MSM and inform an HIVST intervention in three sites in Kenya. Community researchers in each site selected eight seeds to complete a demographic form and network surveys for 15 each of their sexual and social network members. Seeds recruited three respondents, including two regular service users and one MSM who was “unreached” by the program, who then each identified three respondents, resulting with data on 290 individuals.

**Results:**

Findings illustrate the interconnectedness of community-based organization (CBO) members and non-members. In networks where a majority of members had a CBO membership, members had better contacts with programs and were more likely to have accessed health services. Larger networks had more HIV testing and seeds with frequent testing had a positive influence on their network members also being tested frequently. HIVST was tried in very few networks. Almost all network members were willing to use HIVST.

**Conclusion:**

Willingness to use HIVST was nearly universal and points to the importance of networks for reaching individuals not enrolled in programs. Network analysis can help in understanding which type of networks had higher testing and how network-based approaches can be useful to promote HIVST in certain contexts.

## Introduction

UNAID’s “ambitious” 90-90-90 target calls for the provision of HIV treatment “to all who need it” [1]. The target has since been accelerated to testing, treating, and virally suppressing HIV among 95% of people living with the virus to “end the AIDS epidemic by 2030” [2]. To achieve these targets, questions remain about how to reach individuals unconnected to testing and treatment services. HIV self-testing (HIVST) has been promoted as a novel way of reaching undiagnosed populations. In 2016, the World Health Organization (WHO) released new guidelines to “[s]upport the implementation and scale-up of ethical, effective, acceptable and evidence-based approaches to HIVST” [3]. HIVST has been touted as a strategy for reaching individuals who may be less likely to access HIV testing in health care centres. This includes men, who are often less likely to access HIV testing services [4, 5], as well as men who have sex with men (MSM), who experience further barriers due to stigmatization and criminalization in many settings globally [6, 7]. Evidence from a number of studies [8–10] and systematic reviews [11, 12] across populations have found HIVST to be feasible, acceptable, and accurate. A community-based HIVST study conducted in rural Malawi successfully reached men, younger age groups, and some individuals considered to be “at-risk” of acquiring HIV [13]. Men in studies in Sub-Saharan Africa have shown a willingness to use HIVST [9, 10, 14, 15], including in Kenya [8, 16]. A systematic review specifically among MSM found that HIVST could increase the testing frequency and reach first-time or infrequent testers [17]. Despite high levels of acceptance and convenience, evidence from another systematic review on men’s perceptions of HIVST in Sub-Saharan Africa also pointed to challenges in pre-and post-test counselling, mistrust over the accuracy of results, and questions as to where men will collect HIVST, raising the need for community-level education campaigns to maximize reach [18].

While “End of AIDS” discourse has brought with it a prioritization of biomedical technological approaches to addressing HIV, such as HIVST, these testing and treatment targets struggle to be met globally, posing the question of what is lost with a narrowing focus towards biomedical solutions [19]. Baral et al.’s modified social-ecological model to guide HIV research among key populations, including MSM, examines multi-level risk environments by situating individual-level variables within networks, communities, policy contexts, and epidemic environments, highlighting the importance of data that explores multiple levels of risk [20]. Understanding the social networks of MSM is particularly key to tailoring interventions, such as HIVST, for particular locations [21–24]. This is especially true in stigmatized and criminalized settings [22, 23]. Network analysis sees the social as “intimately entwined with biological factors to form complex systems”,^22,p.iii70^ moving beyond individual-level epidemiological studies towards network analyses. Network data collection, which can be done rapidly with a small number of initial “seeds”, who then bring in their partners and so-forth, can unveil patterns typically hidden in aggregate-level analysis, such as network sizes, mixing patterns, and characteristics of sexual networks [24]. Findings from network analysis have great potential to inform who would benefit most from program interventions [23]. In their work exploring the sexual networks of MSM with men in southern India, Lorway et al. demonstrate how sexual network data, in conjunction with behavioural and ethnographic data, can yield specific insights into where interventions can be prioritized [22].

In addition to understanding disease transmission, networks play an important role in sharing information about new HIV prevention technologies [25]. A review of network studies among MSM has shown that network members share similar norms, attitudes, and HIV risk behaviours, and that network-based interventions can be an important means of addressing risk behaviours and increasing HIV testing [26]. The review suggests that network membership might be a stronger predictor of risk behaviours than individual-level characteristics, as members of networks share information and support and influence attitudes and behaviours [26]. While there is a gap in research exploring the distribution of HIVST through the social and sexual networks of MSM, a few studies have pointed to the feasibility of this approach [27–29], as well as its potential for reaching MSM who have not previously tested for HIV [30].

As part of a larger mixed-methods study to evaluate the benefit of the community-based implementation of HIVST strategies, we conducted network mapping in partnership with community-based programs among MSM in three counties in Kenya. Specifically, we were interested in understanding how network depictions can illustrate findings that might be hidden by relying on aggregated survey data alone and how network assessments can be an important tool to highlight similarities and differences across sites to inform the implementation of the HIVST intervention.

## Methods

### Study setting

As part of a larger study to evaluate the impact of a community-based implementation of HIVST delivery strategies among MSM in Kenya (for details see [31]), we undertook a socio-sexual network study to characterize and identify patterns of connection between different types of service using (or avoiding) MSM in each of three study sites (Kisumu, Mombasa, Kiambu counties). The study was implemented by the University of Manitoba and Partners for Health and Development in Africa (PHDA), in partnership with the National AIDS and STI Control Programme (NASCOP), as well as community-based partners G10 (an MSM research network in Kenya), and three community-based organizations (CBOs) in Kenya: Mamboleo Peer Empowerment Group (MPEG in Kiambu), Men Against AIDS Youth Group (MAAYGO in Kisumu), and the HIV & AIDS People’s Alliance of Kenya (HAPA Kenya in Mombasa).

In terms of population composition, Kiambu has a slightly higher population (1.6 million), compared to Kisumu and Mombasa (1 million each). HIV prevalence in the general population in Kisumu is 16%, and 4% in both Mombasa and Kiambu [32], compared to the self-reported HIV prevalence among MSM of 13%, 19% and 23% in the same three sites [33]. According to size estimation studies of physical hotspot spaces where MSM meet sexual partners, there were an estimated 2,492 MSM in Kisumu, 2,855 MSM in Mombasa, and 1,664 MSM in Kiambu [34]. However, these counts underestimate the population size, as internet-based mapping has revealed that 25% of MSM seeking sexual partners do not visit physical hotspots [35]. According to Kenya key populations program data, 53% of the estimated population of MSM living with HIV were known and registered in key population programs at the end of 2018 [36].

### Data Collection

Following a community-based research methods approach [37], training of community researchers (CRs) took place over two days in March 2019. Four CRs were recruited from each study site. The training included sessions on HIVST, research ethics [38], and the process for administering the socio-sexual network survey. During the training, the CRs reviewed and finalized the data collection tools.

Data collection occurred between March and April 2019, for approximately two-and-a-half weeks per site. The CRs in each site selected eight seed respondents (N=24) to complete a demographic form and short network surveys for 15 of their sexual and 15 of their social network members (Appendix Table 2). Sexual network members were defined as partners (either male or female) with whom the seed had sex in the past 12 months. Social network members were defined as MSM contacts with whom respondents had communicated with in the past 30 days. Each CR recruited two seeds, including someone who had accessed services from the programs and someone who had never accessed services from MSM programs. Following the survey, the seeds then selected three individuals from their sexual network contacts to participate in the study: *1 young (18-29 years) service user*; *1 older (30 years and older) service user; and 1 “unreached” MSM who has never accessed services.* The three new respondents each similarly identified three respondents from their network; thus, in addition to the initial seeds, there were 2 waves of recruitment. All participants were 18 years and above, identified as MSM, and had anal or oral sex with another male in the previous 12 months.

The survey included questions on age and social-economic status; the location where a respondent met and/or had sex; whether the person was “out” or “closeted” in their community (i.e., disclosed to family, friends, CBO-based health care providers, married to a woman); means of connecting with other MSM (i.e., social media, cruising/hotspots, CBO events); and whether they were enrolled in an MSM program. For the purpose of the study, MSM program enrollment was self-reported and included enrollment in *any* MSM program.

### Analysis

The network visualizations were depicted to understand how network members share characteristics and to explore whether network approaches might be a useful strategy to reach those individuals not connected to programs and services more effectively. The basic idea behind the visualizations were to understand whether the network members share common characteristics with the seeds and whether seeds can play a role in implementing the HIVST program in the country. The analysis focused on presenting the profile of respondents, visualization and characterization of networks, and uptake of services across three different sites. As HIVST was a relatively new intervention at the time of the study, we further analyzed data related to health services access and HIV testing. For analysis, RDSAT 7.1(Cornell University Ithaca, NY), and Stata 15.0 (Stata Corp, Texas USA) were used. Network diagrams were created using NetDraw 2.1 (NetDraw Software for Network Visualization, Lexington, NY) to understand the size of the MSM network, identify patterns of connection between different sub-populations of MSM, and understand how these connections pivot on characteristics such as age, gender, sexual identity, disclosure, program enrollment, and meeting places for sex. In the bivariate analysis, t-tests were used to test for differences in outcomes across sites.

### Ethics

Ethics approval was obtained from the institutional review boards of the Kenyatta National Hospital – University of Nairobi, Kenya (P557/08/2018) and the University of Manitoba – Health Research Ethics Board, Canada (HS22205).

## Results

### Profile of the seeds and their immediate contacts

Table 1 shows the profile of the seeds and their immediate contacts (network members) which resulted in data on 290 individuals. Of these 290 individuals, about 29% were from Mombasa and 35% were each from Kisumu and Kiambu. Overall, the median age was 26 years. Ninety-eight percent of participants identified as men and 2% identified as either a woman or both as a man and a woman. Of the total sample, 77% of participants identified as gay and 22% as bisexual. Sixty percent of the seeds and their network members were single, 35% were in a committed relationship with a man, and 7% were in a committed relationship with a woman. While 78% of participants revealed their sexual identity to other community members at hotspots and 48% with health care workers at a CBO, only 13% of individuals disclosed their identity to family members, relatives, or neighbours in their community. Sixty-seven percent of the seeds and network members received and 52% paid money or gifts in exchange for sex in the last 3 months preceding the survey.

**Table 1:** Demographic profile of the seeds and immediate network members: Overall and site-specifics

Characteristics	Overall	Sites		
		Mombasa	Kisumu	Kiambu
**N**	**290**	**83**	**103**	**104**
**Age**				
15-19	7.6	18.1	1.0	5.8
20-24	34.8	21.7	27.2	52.9
25-29	23.1	20.5	28.2	20.2
30 and above	34.5	39.8	43.7	21.2
Median age (IQR)	26.0 (22.0-28.0)	27.0 (24.0-29.0)	28.0 (24.0-29.0)	24.0 (22.0-26.0)
**Gender**				
Man	98.3	96.4	100.0	98.1
Woman	0.3	0.0	0.0	1.0
Both	1.4	3.6	0.0	1.0
**Sexual identity**				
Straight	1.4	4.8	0.0	0.0
Gay	76.8	78.3	80.4	72.1
Bisexual	21.8	16.9	19.6	27.9
**Sexual behaviour disclosure**				
Open about same sex behaviour to a family member or relative	13.4	14.5	16.5	9.6
Open about same sex behaviour to neighbour and community	12.8	18.1	11.7	9.6
Open about same sex behaviour in the MSM community at hotspot	77.9	79.5	75.7	78.8
Open about same sex behaviour with health care workers at MSM CBO	48.3	42.2	50.5	51.0
No response/None	14.1	4.8	23.3	12.5
**Relationship status**				
Single	60.0	63.9	53.4	63.5
Married (to a woman)	4.1	4.8	2.9	4.8
Committed relationship with a man	35.2	30.1	42.7	31.7
Committed relationship with a woman	7.2	2.4	11.7	6.7
Divorced	2.8	1.2	6.8	0.0
**Percentage who:**				
Received money or gifts in exchange for sex in the last 3 months	67.4	88.0	85.9	33.0
Given money or gifts in exchange of sex in last 3 months	52.2	33.7	86.3	33.7
Currently enrolled in an MSM CBO	59.0	51.2	57.8	66.3
Contacted to access health services in an MSM CBO in last 3 months	58.6	41.0	60.2	71.2
Accessed health services in an MSM CBO in last 3 months^a^	62.1	68.7	58.3	60.6
**Frequency of HIV testing as:**				
Never/Rarely	11.0	3.7	21.2	6.8
Once a year	11.0	17.3	15.2	1.9
More than once a year	78.1	79.0	63.6	91.3
Ever tried HIVST	28.9	40.7	31.3	17.3^$,#^
Willing to try HIVST	93.7	90.4	95.0	95.1

^a^Among those contacted for health services

^$^Different from Mombasa at *p *< 0.001

# Different from Kisumu at *p *< 0.05

Table 1 depicts the profile of the individuals by sites. While the profile of seeds and network members in Mombasa and Kisumu was similar, individuals from Kiambu had a different profile. The seeds/network partners in Kiambu were relatively younger; approximately one-third received or gave money/gifts in exchange of sex in the last 3 months, compared to over 80% in the other two sites; and had the highest level of HIV testing as part of routine HIV care (>90% tested for HIV more than once a year), but had the least exposure to HIVST (just 17% in Kiambu, compared to 41% in Mombasa, and 31% in Kisumu).

**Fig. 1:**
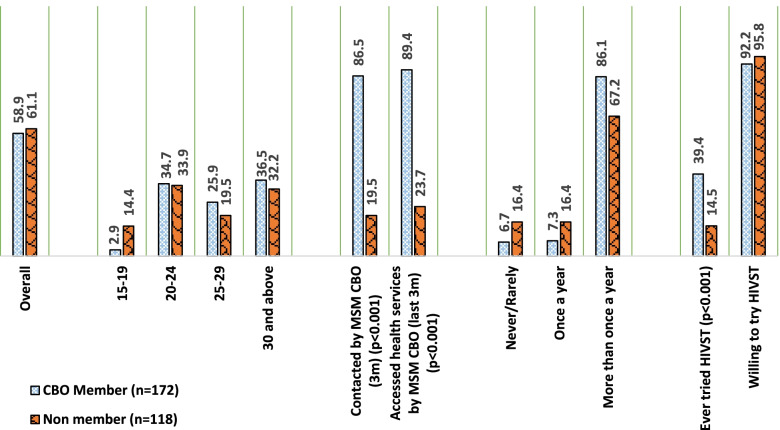
Profile of seeds and initial networks by CBO membership and non-membership

The demographic data also collected information about membership to a CBO and access to health services, including HIV testing and uptake of HIVST. Of the overall sample, about 59% were registered with a CBO. However, of those who were contacted to access health services, only 62% actually accessed services in the 3 months preceding the survey. Results show that while 29% ever tried HIVST, willingness to use HIVST was around 94% among seeds and their networks. The differences in profile and access to health services among individuals by CBO membership is presented in Fig. 1. Among CBO members, a very small proportion (<3%) of MSM were young people aged 15-19 years. Contact with the CBO, access to the health services provided by them, and frequency of HIV testing through the routine program was significantly higher among members versus non-members. Despite HIVST being recently introduced in Kenya, 39% of CBO members had tried HIVST compared to 15% of non-members.

### Understanding network characteristics through network visualization

**Fig. 2:**
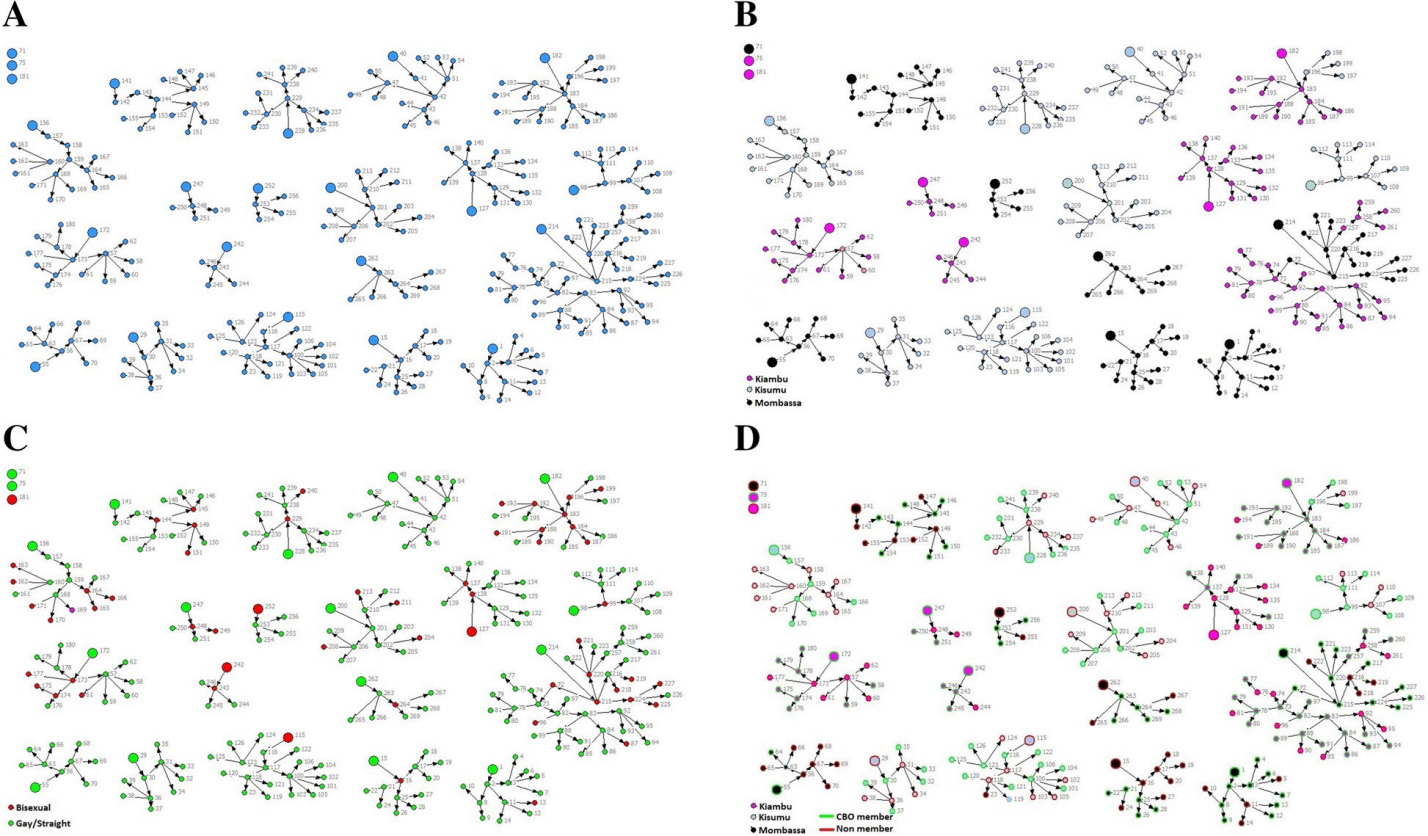
Basic profile of network: Overall by site, sexual identity, and CBO membership

Figure 2a-d provides basic characteristics of the seeds and their networks. Altogether, 19 networks involving 262 seeds were depicted. The networks varied in size with some much larger (e.g., network of seed # 214, 182, 156, 115) than others (e.g., # 242, 247, 252). Moreover, there were three seeds who could not recruit a single member of their network for the study due to some CRs dropping out of the study (Fig. 2a). Networks in Kiambu and Mombasa were of mixed size with an average number of network size of 11 and 12 respectively, whereas Kisumu had networks of moderate size with 7 members on average (Fig. 2b). Data also showed an overlap of socio-sexual networks across sites (i.e., between Mombasa and Kiambu, as well as Kiambu and Kisumu), indicating connections between sites. In terms of sexual identity, most of the networks were among gay men with limited sexual mixing, however, a few gay and bisexual men were also found in the same network. By and large, the sexual orientation of network members matched with the seeds. Figure 2d shows that networks had mixed membership and having a seed who was a CBO member did not necessarily mean greater CBO membership among network members. However, this varied by sites. Importantly, network depictions illustrate that individuals who are not enrolled in CBOs are still closely connected to enrolled members (Descriptive statistics of network maps are presented in Appendix Table 3).

### Access to health services by network members

**Fig. 3:**
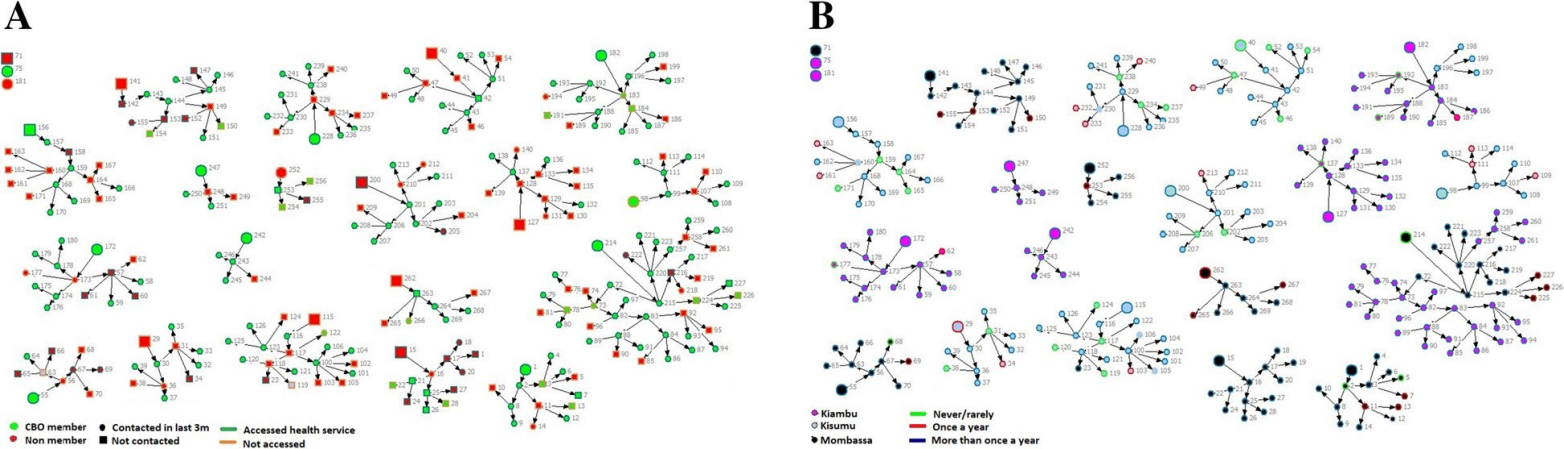
Access to health services in last 3 months among CBO members & non-members and frequency of HIV testing by sites

Network analysis also depicted access to health services, including frequency of HIV testing among network members, and are presented in Fig. 3a-b. Findings showed that where a majority of the network members had a CBO membership, members of those networks had better contacts with programs (87% CBO members versus 20% non-members), and were more likely to have accessed health services in the 3 months preceding the survey (89% members versus 24% non-members). Moreover, seeds having a CBO membership yielded more access to health services by their network members (Fig. 3a). Findings reveal that larger networks had more testing as a large proportion of network members were tested more than once in a year and that a seed being tested frequently had positive influence on their network members also being tested frequently (Fig. 3b). The frequency of HIV testing by routine program appeared to be low in Kisumu, as a relatively higher percent of network members in Kisumu (21%) were never/rarely tested for HIV compared to Mombasa (4%) and Kiambu (7%).

### Previous history and future HIVST willingness

**Fig. 4:**
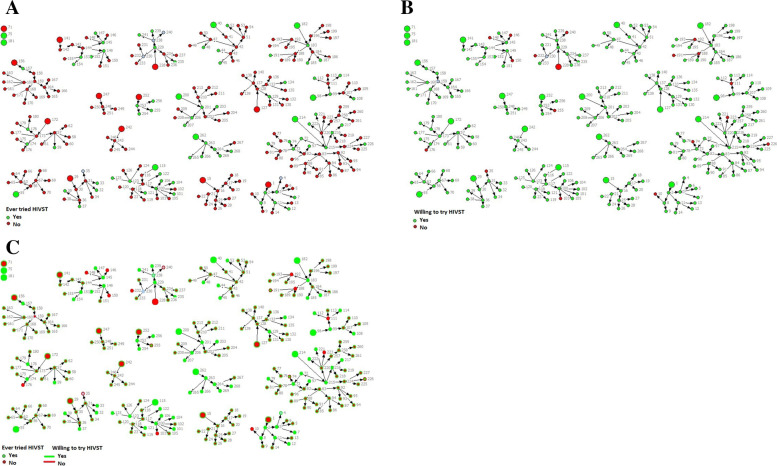
Previous history and future willingness of HIV self-testing among network members

As previously described, only 29% of MSM had ever tried HIVST. Network analysis could help in understanding which type of networks had higher testing and whether network-based approaches can be useful in promoting HIVST going forward. Figure 4a shows that HIVST was tried in very few networks, with some networks reporting ‘zero’ self-testing; additionally, HIVST among network members was not dependent on the seed having used HIVST. Overall, 36% of seeds and 27% of network members had ever tried HIVST. Previous experiences of HIVST were low across the sites (Mombasa- 41%, Kisumu-31%, Kiambu-15%) while relatively higher among those with a CBO membership (39%) compared to non-membership (15%) (network illustration not shown). Contrary to this, almost all network members (93%) were willing to use HIVST in the near future (Fig. 4b). The willingness of future testing was also almost the same among those individuals who were never exposed to HIVST (93% compared to 94% among those who ever used HIVST), especially in Mombasa and Kisumu, where larger network sizes exist (Fig. 4c).

## Discussion

Our findings illustrate differences between geographical sites in ages, sizes of networks, CBO membership, health services access, and HIVST. Our network maps show that networks captured in this study illuminate hidden patterns among networks, as well as the interconnectedness of CBO members and non-members. Willingness to use HIVST was nearly universal and points to the importance of networks for reaching individuals not connected to programs and services. In particular, our analysis highlights the need for different approaches in different contexts. Whereas network-based approaches to HIVST distribution may work well in Mombasa and Kisumu, our findings showed fewer network ties in Kiambu, pointing to potential limitations of a network-based strategy in this site. However, equally important is understanding overlap between networks across sites. Although there were fewer network ties in Kiambu, there were higher rates of testing, pointing to the question of whether active members from Kiambu could be champions of HIVST where there is overlap with members in Kisumu among networks with no HIVST.

Our findings further highlight the important role that communities play in HIV prevention and treatment. In our study, CBO membership had a positive influence on access to health services, with members of networks with more ties to CBOs having more frequent access to services. Similarly, if the seed was a CBO member and had access to health services provided by the CBO, their network members were also likely to have more access to services. Despite the fact that seeds of many networks had accessed HIVST, their network members did not appear to have previous experience with HIVST. Research in Nigeria has demonstrated the feasibility and acceptability in distributing HIVST to MSM through “key opinion leaders” to their networks [39]. Furthermore, concerns over linkage to treatment were addressed through active follow-up and access to community-based clinics [39], both of which are similarly important aspects in our sites. Network distribution has also proven feasible in other sites, such as in South Africa [28], Uganda, [29] China [27], and the United-States [30]. In their updated policy brief, the WHO has called for the engagement of communities in designing HIVST delivery models to support the successful provision of HIVST [40]. While community organizations continue to be defunded and deprioritized, [19] our findings re-emphasize the important role to be played by community organizations and peer outreach workers in not only “ending AIDS”, but in meeting the needs of their members through tailored context-specific responses.

### Limitations

This study has some limitations. First, the analysis was done using few characteristics, such as site, sexual identity, and CBO membership. Although data on other characteristics, like place of solicitation and age were collected, they were not used due to relatively higher missing values. Second, after completing the respondent demographic form, a short survey was administered among 290 members to collect information of about 25-30 members in their social and sexual networks. The information was collected on their socio-demographic and sexual profile, however, it did not include information about their routine access to programs including HIV testing and HIVST. It was difficult to collect this information as the seeds might not know and remember such information for each of their network members. Lastly, we only conducted descriptive analyses and illustrated MSM behaviour in a network form without adjusting for many other programmatic variables that might have a stronger bearing on the uptake of HIVST. Despite these limitations, the present analysis followed a simple and innovative way of depicting the data which could help programs devise strategies to engage “unreached” MSM with various program services. The depiction of heterogeneity in individual characteristics and network dynamics across sites would help in making those strategies specific to certain population groups and geographies.

### Conclusion

Network analysis highlights heterogeneity normally hidden in aggregate survey data. The network depictions uncover important information to help guide program planning and the roll out of new biomedical technologies to achieve urgent and time-sensitive global 95-95-95 targets. Network-based approaches demonstrate the need for community-based and community-led approaches to reaching MSM with these new emerging technologies and ensuring linkages to programs and services in order to achieve the “end of AIDS”. In particular, network analysis can help in understanding which type of networks have higher testing and how network-based approaches can be useful in promoting HIVST in certain contexts.

## Data Availability

The datasets used and analysed during the study will be available from the corresponding author on reasonable request.

## Appendix

Tables 2 and 3

**Table 2:** Recruitment success rate by site

Site	Number of Primary Seeds recruited by CR			Next level of recruitment by seeds (assuming each will recruit 9 respondents of different categories)			Level of recruitment				Respondents in contact list (15 social and 15 sexual network)		
	Expected	Actual	% actual	Expected	Actual	% actual	Level 1	Level 2	Level 3+	N	Expected	Actual	% actual
Mombasa	8	8	100.0	72	72	100.0	33.3	31.9	34.7	72	2160	2,418	111.9
Kisumu	8	8	100.0	72	96	133.3	32.6	32.6	34.7	96	2160	2,673	123.8
Kiambu	8	8	100.0	72	96	133.3	37.9	33.7	28.4	96	2160	3,054	141.4
**Total**	**24**	**24**^**a**^	**100.0**	**216**	**264**	**122.2**	**34.7**	**32.8**	**32.4**	**264**	**6480**	**8,145**	**125.7**

^a^Additional Primary Seeds excluded as they were not able to recruit any respondents, *N* = 290

**Table 3:** Descriptive statistics of network map presented in Fig. 2

Indicator	Overall	Mombasa	Kisumu	Kiambu
Mean network size	9.8 (SD: 8.0)	11.5 (SD: 10.3)	7.3 (SD: 4.1)	10.8 (SD: 8.3)
Gender identity of seeds (%)				
* Bisexual*	23.6	9.1	30.0	33.3
* Gay*	76.6	90.9	70.0	66.7
^**a**^**Percentage of:**				
* Network members with same gender identity as of their seeds*	49.4	53.6	46.4	48.9
* Networks with >80% of seeds and members of same gender identity*	43.7	50.0	66.7	20.0
* Networks with 50-80% of seeds and members of same gender identity*	18.8	25.0	0.0	40.0
* Networks with <50% of seeds and genders of same gender identity*	37.5	25.0	33.3	40.0
CBO membership of seeds (%)				
* Member*	46.7	36.3	30.0	33.3
* Non-member*	53.3	63.7	70.0	66.7
^**a**^**Percentage of:**				
* Network members with CBO membership as of their seeds*	50.9	52.4	49.5	51.1
* Networks with >80% of seeds and members of same membership status*	6.7	0.0	0.0	16.7
* Networks with 50-80% of seeds and members of same membership status*	73.3	100.0	80.0	50.0
* Networks with <50% of seeds and genders of same membership status*	20.0	0.0	20.0	33.3
% of network members who contacted by CBO to access health services in 3 months whose seed was a CBO member	58.9	44.7	73.1	53.8

^a^Among the networks with at least one member. || Sample size: Seeds=24 & Network Members= 240

## Abbreviations

CBO: community-based organization
CR: community researcher
HAPA Kenya: HIV & AIDS People’s Alliance of Kenya
HIVST: HIV self-testing
MAAYGO: Men Against AIDS Youth Group
MPEG: Mamboleo Peer Empowerment Group
MSM: men who have sex with men
NASCOP: National AIDS and STI Control Programme
PHDA: Partners for Health and Development in Africa
WHO: World Health Organization
